# Supplementary material for: Ancylostoma ceylanicum: The Neglected Zoonotic Parasite of Community Dogs in Thailand and Its Genetic Diversity among Asian Countries
Source: Animals (Basel). 2020 Nov 19;10(11):2154. doi: 10.3390/ani10112154 (PMC7699415; doi:10.3390/ani10112154)
Supplement: Supplementary file 1 [file animals-10-02154-s001.zip › Kladkempetch Table S3.docx]

Article

*Ancylostoma ceylanicum*: The Neglected Zoonotic Parasite of Community Dogs in Thailand and Its Genetic Diversity among Asian Countries

Doolyawat Kladkempetch, Sahatchai Tangtrongsup and Saruda Tiwananthagorn

**Table S3.**  Summary of hookworm infection in dog fecal samples and soil samples obtained from 53 temples as assessed by microscopic examination.

| **Province** | **Temple name** | **Number of dog samples** | | **Number of soil samples** | | **Detection of hookworm in** | | **Soil collecting areas** | | | |
| --- | --- | --- | --- | --- | --- | --- | --- | --- | --- | --- | --- |
|  |  | **examined** | **positive** | **examined** | **positive** | **Dog** | **Soil** | **Temple courtyard** | **Dog dwelling area** | **Human activity area** | **Under big tree** |
| Chiang Mai | Wat Siri Mangkalajarn | 5 | 0 | 4 | 0 | no | no | no | no | no | no |
|  | Wat Ban Rai Pattanaram | 4 | 0 | 4 | 0 | no | no | no | no | no | no |
|  | Wat Phra That Doi Saket | 5 | 1 | 4 | 2 | yes | yes | no | yes | yes | no |
|  | Wat Ban Huai Bon | 7 | 6 | 4 | 1 | yes | yes | no | yes | no | no |
|  | Wat Mae Hong Khrai | 4 | 1 | 4 | 0 | yes | no | no | no | no | no |
|  | Wat Sahakon | 7 | 2 | 4 | 0 | yes | no | no | no | no | no |
|  | Wat Chaiya Sathan | 7 | 3 | 4 | 1 | yes | yes | no | yes | no | no |
|  | Wat Pra Nawn | 5 | 3 | 4 | 2 | yes | yes | yes | no | no | yes |
|  | Wat Mae Rim | 4 | 2 | 4 | 0 | yes | no | no | no | no | no |
|  | Wat Pa Dara Phirom | 6 | 2 | 4 | 0 | yes | no | no | no | no | no |
|  | Wat Pa Nam Rin | 5 | 2 | 4 | 0 | yes | no | no | no | no | no |
|  | Wat Makham | 4 | 3 | 4 | 1 | yes | yes | no | no | no | yes |
|  | Wat Hnong Pahn Jieng | 6 | 2 | 4 | 1 | yes | yes | no | no | no | yes |
|  | Wat Ban Phae | 4 | 0 | 4 | 1 | no | yes | no | yes | no | no |
|  | Wat Tha Kham | 5 | 2 | 4 | 0 | yes | no | no | no | no | no |
|  | Wat Santitham | 4 | 0 | 4 | 0 | no | no | no | no | no | no |
|  | Wat Don Chan | 8 | 7 | 4 | 0 | yes | no | no | no | no | no |
|  | Wat Pa Ngio | 4 | 0 | 4 | 2 | no | yes | no | yes | yes | no |
|  | Wat Puak Pia | 2 | 0 | 4 | 1 | no | yes | no | no | yes | no |
|  | Wat Muang Sat Luang | 5 | 0 | 4 | 0 | no | no | no | no | no | no |
|  | Wat San Pa Liang | 5 | 0 | 4 | 0 | no | no | no | no | no | no |
|  | Wat Rong San | 5 | 0 | 4 | 0 | no | no | no | no | no | no |
|  | Wat Suphan Rangsi | 4 | 0 | 4 | 0 | no | no | no | no | no | no |
|  | Wat Nam Bo Luang | 7 | 6 | 4 | 0 | yes | no | no | no | no | no |
|  | Wat San Klang | 8 | 3 | 4 | 3 | yes | yes | yes | yes | yes | no |
|  | Wat Pong Chang Khot | 4 | 4 | 4 | 1 | yes | yes | yes | no | no | no |
|  | Wat Huai Kiang | 4 | 0 | 4 | 0 | no | no | no | no | no | no |
|  | Wat Kuna Nu-sawn | 9 | 8 | 4 | 0 | yes | no | no | no | no | no |
|  | Wat Wiwek Wanaram | 7 | 0 | 4 | 0 | no | no | no | no | no | no |
|  | Wat Weluwan | 5 | 0 | 4 | 0 | no | no | no | no | no | no |
| Chiang Rai | Wat Phrathat Pha Ngao | 3 | 0 | 4 | 0 | no | no | no | no | no | no |
|  | Wat Chedi Luang | 7 | 0 | 4 | 0 | no | no | no | no | no | no |
|  | Wat Bencha Pattana | 6 | 0 | 4 | 1 | no | yes | no | no | yes | no |
|  | Wat Maesai | 5 | 0 | 4 | 0 | no | no | no | no | no | no |
|  | Wat Muang Daeng | 7 | 0 | 4 | 0 | no | no | no | no | no | no |
|  | Wat Fang Min | 9 | 0 | 4 | 0 | no | no | no | no | no | no |
|  | Wat San Sai Luang | 7 | 0 | 4 | 0 | no | no | no | no | no | no |
|  | Wat Si Bunruang | 5 | 0 | 4 | 0 | no | no | no | no | no | no |
|  | Wat Pong Sali | 2 | 0 | 4 | 0 | no | no | no | no | no | no |
|  | Wat Rong Than | 3 | 0 | 4 | 0 | no | no | no | no | no | no |
|  | Wat Si Sutthawat | 9 | 3 | 4 | 0 | yes | no | no | no | no | no |
|  | Wat Fuey Hai | 7 | 3 | 4 | 0 | yes | no | no | no | no | no |
| Lampang | Wat Phra Chedi Sao | 8 | 7 | 4 | 0 | yes | no | no | no | no | no |
|  | Wat Wangphrao | 4 | 0 | 4 | 0 | no | no | no | no | no | no |
|  | Wat Phrabat Mae Thai | 6 | 2 | 4 | 3 | yes | yes | no | yes | yes | yes |
|  | Wat Pong Sanuk Tai | 2 | 0 | 4 | 0 | no | no | no | no | no | no |
| Phayao | Wat Kiew Chumpoo | 6 | 1 | 4 | 0 | yes | no | no | no | no | no |
|  | Wat Si Ku Iang | 7 | 1 | 4 | 0 | yes | no | no | no | no | no |
|  | Wat Huai Yang Kham | 7 | 2 | 4 | 2 | yes | yes | no | yes | no | yes |
|  | Wat Bunyong | 5 | 0 | 4 | 0 | no | no | no | no | no | no |
|  | Wat Analayo | 9 | 3 | 4 | 0 | yes | no | no | no | no | no |
|  | Wat Rong Pa Pao | 9 | 0 | 4 | 0 | no | no | no | no | no | no |
|  | Wat Dok Bua | 7 | 0 | 4 | 0 | no | no | no | no | no | no |
| Total | 53 | 299 | 79 | 212 | 22 | 25 | 14 | 3 | 8 | 6 | 5 |
|  | % |  | 26.42 |  | 10.38 | 47.17 | 26.42 | 5.66 | 15.09 | 11.32 | 9.43 |

**Publisher’s Note:** MDPI stays neutral with regard to jurisdictional claims in published maps and institutional affiliations.

| 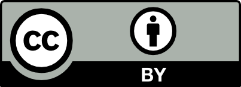 | © 2020 by the authors. Licensee MDPI, Basel, Switzerland. This article is an open access article distributed under the terms and conditions of the Creative Commons Attribution (CC BY) license (http://creativecommons.org/licenses/by/4.0/). |
| --- | --- |
