# Supplementary material for: Ancylostoma ceylanicum: The Neglected Zoonotic Parasite of Community Dogs in Thailand and Its Genetic Diversity among Asian Countries
Source: Animals (Basel). 2020 Nov 19;10(11):2154. doi: 10.3390/ani10112154 (PMC7699415; doi:10.3390/ani10112154)
Supplement: Supplementary file 1 [file animals-10-02154-s001.zip › Kladkempetch Table S4.docx]

Article

*Ancylostoma ceylanicum*: The Neglected Zoonotic Parasite of Community Dogs in Thailand and Its Genetic Diversity among Asian Countries

Doolyawat Kladkempetch, Sahatchai Tangtrongsup and Saruda Tiwananthagorn

**Table S4.** Hookworm detection in the soil based on the site of collection in temples in Thailand.

| **Site of Collection** | **Examined (n)** | **Positive (%)** | **OR (95% CI)** | **P value** |
| --- | --- | --- | --- | --- |
| Temple courtyard | 53 | 3 (5.66) | ref | |
| Dog dwelling area | 53 | 8 (15.09) | 2.96 (0.74-11.86) | 0.125 |
| Human activity area | 53 | 6 (11.32) | 2.13 (0.5-8.99) | 0.305 |
| Under the big tree | 53 | 5 (9.43) | 1.74 (0.39-7.67) | 0.467 |
| Total | 212 | 22 |  |  |

**Publisher’s Note:** MDPI stays neutral with regard to jurisdictional claims in published maps and institutional affiliations.

| 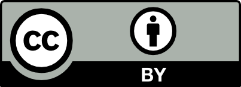 | © 2020 by the authors. Licensee MDPI, Basel, Switzerland. This article is an open access article distributed under the terms and conditions of the Creative Commons Attribution (CC BY) license (http://creativecommons.org/licenses/by/4.0/). |
| --- | --- |
