# Supplementary material for: Ancylostoma ceylanicum: The Neglected Zoonotic Parasite of Community Dogs in Thailand and Its Genetic Diversity among Asian Countries
Source: Animals (Basel). 2020 Nov 19;10(11):2154. doi: 10.3390/ani10112154 (PMC7699415; doi:10.3390/ani10112154)
Supplement: Supplementary file 1 [file animals-10-02154-s001.zip › Kladkempetch Table S5.docx]

Article

*Ancylostoma ceylanicum*: The Neglected Zoonotic Parasite of Community Dogs in Thailand and Its Genetic Diversity among Asian Countries

Doolyawat Kladkempetch, Sahatchai Tangtrongsup and Saruda Tiwananthagorn

**Table S5.** Haplotypes of *Ancylostoma* hookworms based on the *cox1* gene in Thailand based on sampling location.

| **Hookworm species** | ***cox1* haplotype** | **Number of sequences** | **Province** | **Isolation no.** | **Source** | **Accession no.** |
| --- | --- | --- | --- | --- | --- | --- |
| ***A. ceylanicum*** | Acy-COX1-TH01 | 21 | Chiang Mai | CM11 | Dog | LC533318 |
|  |  |  |  | CM20 |  |  |
|  |  |  |  | CM31 |  |  |
|  |  |  |  | CM32 |  |  |
|  |  |  |  | CM34 |  |  |
|  |  |  |  | CM37 |  |  |
|  |  |  |  | CM68 |  |  |
|  |  |  |  | CM80 |  |  |
|  |  |  |  | CM81 |  |  |
|  |  |  |  | CM105 |  |  |
|  |  |  |  | CM108 |  |  |
|  |  |  |  | CM134 |  |  |
|  |  |  |  | CM137 |  |  |
|  |  |  | Chiang Rai | CR49 | Dog |  |
|  |  |  |  | CR50 |  |  |
|  |  |  | Lampang | LP2 | Dog |  |
|  |  |  |  | LP8 |  |  |
|  |  |  |  | LP9 |  |  |
|  |  |  | Phayao | PY45 | Dog |  |
|  |  |  |  | PY48 |  |  |
|  |  |  |  | PY49 |  |  |
|  |  | 2 | Phayao | PY12 | Soil | LC533327 |
|  |  |  |  | PY72 |  |  |
|  | Acy-COX1-TH02 | 1 | Chiang Mai | CM110 | Dog | LC533319 |
|  | Acy-COX1-TH03 | 1 | Chiang Mai | CM139 | Dog | LC533320 |
|  | Acy-COX1-TH04 | 1 | Chiang Mai | CM144 | Dog | LC533321 |
|  | Acy-COX1-TH05 | 1 | Chiang Mai | CM24 | Dog | LC533322 |
|  | Acy-COX1-TH06 | 1 | Chiang Mai | CM46 | Dog | LC533323 |
|  | Acy-COX1-TH07 | 1 | Chiang Rai | CR48 | Dog | LC533324 |
|  | Acy-COX1-TH08 | 1 | Lampang | LP1 | Dog | LC533325 |
|  | Acy-COX1-TH09 | 1 | Lampang | LP3 | Dog | LC533326 |
|  | Total | 31 |  |  |  |  |
| ***A. caninum*** | Aca-COX1-TH01 | 1 | Chiang Mai | CM06 | Dog | LC533328 |
|  | Total | 1 |  |  |  |  |

**Publisher’s Note:** MDPI stays neutral with regard to jurisdictional claims in published maps and institutional affiliations.

| 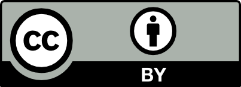 | © 2020 by the authors. Licensee MDPI, Basel, Switzerland. This article is an open access article distributed under the terms and conditions of the Creative Commons Attribution (CC BY) license (http://creativecommons.org/licenses/by/4.0/). |
| --- | --- |
