# Supplementary material for: Ancylostoma ceylanicum: The Neglected Zoonotic Parasite of Community Dogs in Thailand and Its Genetic Diversity among Asian Countries
Source: Animals (Basel). 2020 Nov 19;10(11):2154. doi: 10.3390/ani10112154 (PMC7699415; doi:10.3390/ani10112154)
Supplement: Supplementary file 1 [file animals-10-02154-s001.zip › Kladkempetch Table S1.docx]

Article

*Ancylostoma ceylanicum*: The Neglected Zoonotic Parasite of Community Dogs in Thailand and Its Genetic Diversity among Asian Countries

Doolyawat Kladkempetch, Sahatchai Tangtrongsup and Saruda Tiwananthagorn

**Table S1.** Reference *Ancylostoma cox1* gene sequences used for phylogenetic analysis.

| *Ancylostoma* spp. | Country | Host | Accession no. | References |
| --- | --- | --- | --- | --- |
| *A. duodenale* | China | Human | NC003415 | [1] |
| *A. caninum* | Australia | Dog | NC012309 | [2] |
| *A. ceylanicum* | Cambodia | Human | KF896597 | [3] |
|  |  | Human | KF896599 |  |
|  |  | Human | KF896603 |  |
|  |  | Human | KF896604 |  |
|  |  | Human | KF896605 |  |
|  |  | Dog | KF896596 |  |
|  |  | Dog | KF896598 |  |
|  |  | Dog | KF896600 |  |
|  |  | Dog | KF896602 |  |
|  | China | Cat | KP072069 | [4] |
|  |  | Cat | KP072070 |  |
|  |  | Cat | KP072071 |  |
|  |  | Cat | KP072073 |  |
|  |  | Cat | KP072075 |  |
|  |  | Cat | KP072076 |  |
|  |  | Cat | KP072077 |  |
|  |  | Cat | KP072079 |  |
|  |  | Cat | KP072080 |  |
|  | Japan | Human | LC271155 | [5] |
|  |  | Human | LC271184 |  |
|  | Malaysia | Human | MK792828 | Mohd-Shaharuddin et al., unpublished results |
|  |  | Human | MK792829 |  |
|  |  | Dog | MK792824 |  |
|  |  | Cat | MK792825 |  |
|  |  | Human | KC247737 | [6] |
|  |  | Human | KC247740 |  |
|  |  | Human | KC247744 |  |
|  |  | Dog | KC247729 |  |
|  |  | Dog | KC247734 |  |
|  |  | Dog | KC247742 |  |
|  |  | Cat | KC247728 |  |
|  |  | Cat | KC247743 |  |
|  | Papua  New  Guinea | Human | LC036568 | [5] |
|  | Australia | Human | AJ407937 | [7] |
|  | Tanzania | Dog | MG914068 | [8] |
|  |  | Dog | MG914069 |  |
|  |  | Dog | MG914070 |  |
|  |  | Dog | MG914072 |  |
|  | Thailand | Dog | KF896595 | [3] |

References

1. Hu, M.; Chilton, N.B.; Gasser, R.B. The mitochondrial genomes of the human hookworms, *Ancylostoma duodenale* and *Necator americanus* (Nematoda: Secernentea). *Int J Parasitol* **2002**, *32*, 145–158, doi:10.1016/s0020-7519(01)00316-2.

2. Hu, M.; Chilton, N.B.; Zhu, X.; Gasser, R.B. Single-strand conformation polymorphism-based analysis of mitochondrial cytochrome *c* oxidase subunit 1 reveals significant substructuring in hookworm populations. *Electrophoresis* **2002**, *23*, 27–34, doi:10.1002/1522-2683(200201)23:1<27::AID-ELPS27>3.0.CO;2-7.

3. Inpankaew, T.; Schar, F.; Dalsgaard, A.; Khieu, V.; Chimnoi, W.; Chhoun, C.; Sok, D.; Marti, H.; Muth, S.; Odermatt, P., et al. High prevalence of *Ancylostoma ceylanicum* hookworm infections in humans, Cambodia, 2012. *Emerg Infect Dis* **2014**, *20*, 976–982, doi:10.3201/eid2006.131770.

4. Hu, W.; Yu, X.G.; Wu, S.; Tan, L.P.; Song, M.R.; Abdulahi, A.Y.; Wang, Z.; Jiang, B.; Li, G.Q. Levels of *Ancylostoma* infections and phylogenetic analysis of *cox1* gene of *A. ceylanicum* in stray cat faecal samples from Guangzhou, China. *J Helminthol* **2016**, *90*, 392–397, doi:10.1017/S0022149X15000413.

5. Yoshikawa, M.; Ouji, Y.; Hirai, N.; Nakamura-Uchiyama, F.; Yamada, M.; Arizono, N.; Akamatsu, N.; Yoh, T.; Kaya, D.; Nakatani, T., et al. *Ancylostoma ceylanicum*, novel etiological agent for traveler's diarrhea-report of four Japanese patients who returned from southeast Asia and Papua New Guinea. *Trop Med Health* **2018**, *46*, 6, doi:10.1186/s41182-018-0087-8.

6. Ngui, R.; Mahdy, M.A.; Chua, K.H.; Traub, R.; Lim, Y.A. Genetic characterization of the partial mitochondrial cytochrome oxidase *c* subunit I *(cox1)* gene of the zoonotic parasitic nematode, *Ancylostoma ceylanicum* from humans, dogs and cats. *Acta Trop* **2013**, *128*, 154–157, doi:10.1016/j.actatropica.2013.06.003.

7. Jex, A.R.; Waeschenbach, A.; Hu, M.; van Wyk, J.A.; Beveridge, I.; Littlewood, D.T.; Gasser, R.B. The mitochondrial genomes of *Ancylostoma caninum* and *Bunostomum phlebotomum*--two hookworms of animal health and zoonotic importance. *BMC Genomics* **2009**, *10*, 79, doi:10.1186/1471-2164-10-79.

8. Merino-Tejedor, A.; Nejsum, P.; Mkupasi, E.M.; Johansen, M.V.; Olsen, A. Molecular identification of zoonotic hookworm species in dog faeces from Tanzania. *J Helminthol* **2019**, *93*, 313–318, doi:10.1017/S0022149X18000263.

**Publisher’s Note:** MDPI stays neutral with regard to jurisdictional claims in published maps and institutional affiliations.

| 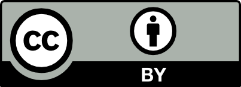 | © 2020 by the authors. Licensee MDPI, Basel, Switzerland. This article is an open access article distributed under the terms and conditions of the Creative Commons Attribution (CC BY) license (http://creativecommons.org/licenses/by/4.0/). |
| --- | --- |
