# Supplementary material for: Ancylostoma ceylanicum: The Neglected Zoonotic Parasite of Community Dogs in Thailand and Its Genetic Diversity among Asian Countries
Source: Animals (Basel). 2020 Nov 19;10(11):2154. doi: 10.3390/ani10112154 (PMC7699415; doi:10.3390/ani10112154)
Supplement: Supplementary file 1 [file animals-10-02154-s001.zip › Kladkempetch Table S2.docx]

Article

*Ancylostoma ceylanicum*: The Neglected Zoonotic Parasite of Community Dogs in Thailand and Its Genetic Diversity among Asian Countries

Doolyawat Kladkempetch, Sahatchai Tangtrongsup and Saruda Tiwananthagorn

**Table S2.** List of *A.* c*eylanicum* haplotypes based on a 259-bp fragment of the *cox1* gene by countries for population analysis.

| Country | Haplotype name | Number of sequences | Accession no. | References |
| --- | --- | --- | --- | --- |
| Thailand | Acy-COX1-TH01 | 21 (from dogs in this study) | LC533318 | In this study |
|  |  | 2 (from soil in this study) | LC533327 |  |
|  |  | 1 | KF896595 | [1] |
|  | Acy-COX1-TH02 | 1 | LC533319 | In this study |
|  | Acy-COX1-TH03 | 1 | LC533320 |  |
|  | Acy-COX1-TH04 | 1 | LC533321 |  |
|  | Acy-COX1-TH05 | 1 | LC533322 |  |
|  | Acy-COX1-TH06 | 1 | LC533323 |  |
|  | Acy-COX1-TH07 | 1 | LC533324 |  |
|  | Acy-COX1-TH08 | 1 | LC533325 |  |
|  | Acy-COX1-TH09 | 1 | LC533326 |  |
| Cambodia | CA01 | 2 | KF896596 | [1] |
|  |  |  | KF896597 |  |
|  | CA02 | 2 | KF896598 |  |
|  |  |  | KF896599 |  |
|  | CA03 | 2 | KF896600 |  |
|  |  |  | KF896601 |  |
|  | CA04 | 2 | KF896602 |  |
|  |  |  | KF896603 |  |
|  | CA05 | 1 | KF896604 |  |
|  | CA06 | 1 | KF896605 |  |
| China | CH01 | 1 | KP072069 | [2] |
|  | CH02 | 1 | KP072070 |  |
|  | CH03 | 3 | KP072071 |  |
|  |  |  | KP072072 |  |
|  |  |  | KP072074 |  |
|  | CH04 | 1 | KP072073 |  |
|  | CH05 | 1 | KP072075 |  |
|  | CH06 | 1 | KP072076 |  |
|  | CH07 | 2 | KP072077 |  |
|  |  |  | KP072078 |  |
|  | CH08 | 1 | KP072079 |  |
|  | CH09 | 1 | KP072080 |  |
| Malaysia | MA01 | 12 | MK792829 | Mohd-Shaharuddin et al., unpublished results |
|  |  |  | MK792830 |  |
|  |  |  | MK792831 |  |
|  |  |  | MK792832 |  |
|  |  |  | MK792833 |  |
|  |  |  | MK792834 |  |
|  |  |  | MK792835 |  |
|  |  |  | KC247734 | [3] |
|  |  |  | KC247735 |  |
|  |  |  | KC247736 |  |
|  |  |  | KC247737 |  |
|  |  |  | KC247738 |  |
|  | MA02 | 22 | MK792814 | Mohd-Shaharuddin et al., unpublished results |
|  |  |  | MK792815 |  |
|  |  |  | MK792816 |  |
|  |  |  | MK792817 |  |
|  |  |  | MK792818 |  |
|  |  |  | MK792819 |  |
|  |  |  | MK792820 |  |
|  |  |  | MK792821 |  |
|  |  |  | MK792822 |  |
|  |  |  | MK792823 |  |
|  |  |  | MK792824 |  |
|  |  |  | MK792825 |  |
|  |  |  | MK792826 |  |
|  |  |  | MK792827 |  |
|  |  |  | MK792828 |  |
|  |  |  | KC247727 | [3] |
|  |  |  | KC247728 |  |
|  |  |  | KC247729 |  |
|  |  |  | KC247730 |  |
|  |  |  | KC247731 |  |
|  |  |  | KC247732 |  |
|  |  |  | KC247733 |  |
|  | MA03 | 3 | KC247739 |  |
|  |  |  | KC247740 |  |
|  |  |  | KC247741 |  |
|  | MA04 | 2 | KC247742 |  |
|  |  |  | KC247743 |  |
|  | MA05 | 1 | KC247744 |  |

References

1. Inpankaew, T.; Schar, F.; Dalsgaard, A.; Khieu, V.; Chimnoi, W.; Chhoun, C.; Sok, D.; Marti, H.; Muth, S.; Odermatt, P., et al. High prevalence of *Ancylostoma ceylanicum* hookworm infections in humans, Cambodia, 2012. *Emerg Infect Dis* **2014**, *20*, 976–982, doi:10.3201/eid2006.131770.

2. Hu, W.; Yu, X.G.; Wu, S.; Tan, L.P.; Song, M.R.; Abdulahi, A.Y.; Wang, Z.; Jiang, B.; Li, G.Q. Levels of *Ancylostoma* infections and phylogenetic analysis of *cox1* gene of *A. ceylanicum* in stray cat faecal samples from Guangzhou, China. *J Helminthol* **2016**, *90*, 392–397, doi:10.1017/S0022149X15000413.

3. Ngui, R.; Mahdy, M.A.; Chua, K.H.; Traub, R.; Lim, Y.A. Genetic characterization of the partial mitochondrial cytochrome oxidase *c* subunit I *(cox1)* gene of the zoonotic parasitic nematode, *Ancylostoma ceylanicum* from humans, dogs and cats. *Acta Trop* **2013**, *128*, 154–157, doi:10.1016/j.actatropica.2013.06.003.

**Publisher’s Note:** MDPI stays neutral with regard to jurisdictional claims in published maps and institutional affiliations.

| 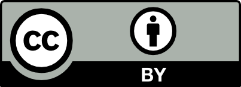 | © 2020 by the authors. Licensee MDPI, Basel, Switzerland. This article is an open access article distributed under the terms and conditions of the Creative Commons Attribution (CC BY) license (http://creativecommons.org/licenses/by/4.0/). |
| --- | --- |
